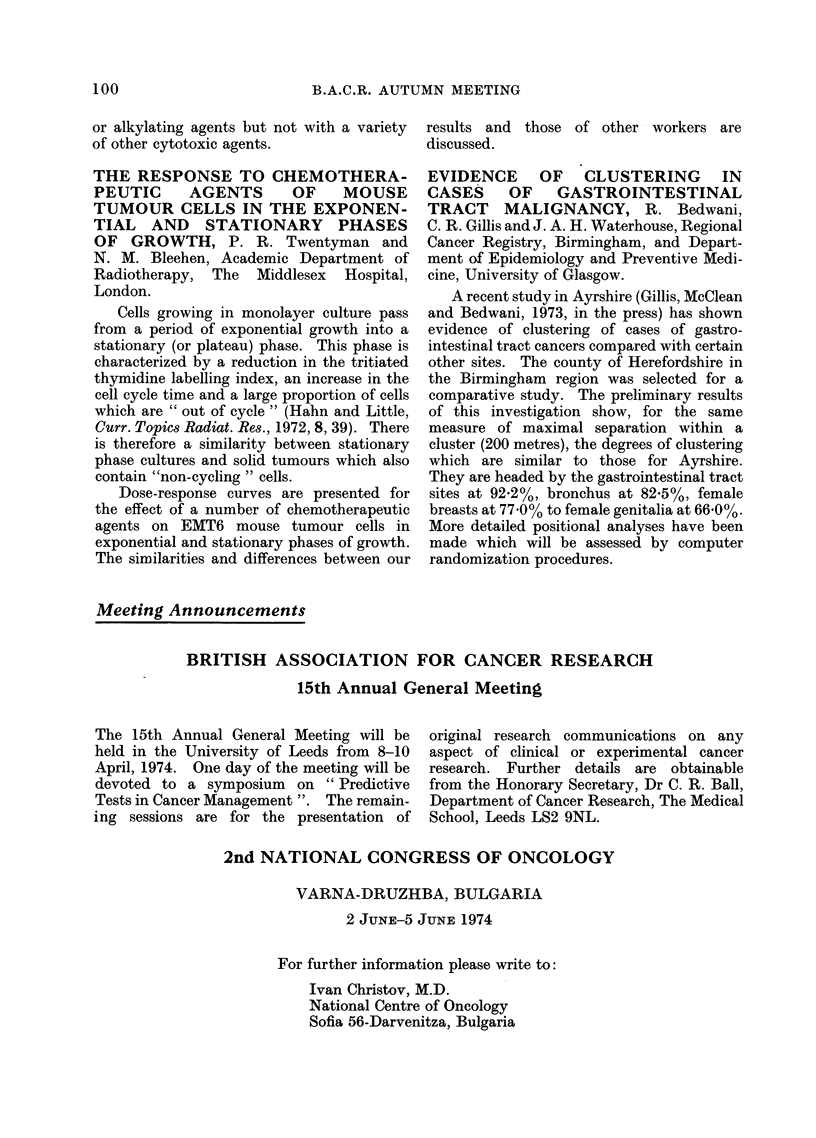# Proceedings: The response to chemotherapeutic agents of mouse tumour cells in the exponential and stationary phases of growth.

**DOI:** 10.1038/bjc.1974.40

**Published:** 1974-01

**Authors:** P. R. Twentyman, N. M. Bleehen


**THE RESPONSE TO CHEMOTHERAPEUTIC AGENTS OF MOUSE TUMOUR CELLS IN THE EXPONENTIAL AND STATIONARY PHASES OF GROWTH,** P. R. Twentyman and N. M. Bleehen, Academic Department of Radiotherapy, The Middlesex Hospital, London.

Cells growing in monolayer culture pass from a period of exponential growth into a stationary (or plateau) phase. This phase is characterized by a reduction in the tritiated thymidine labelling index, an increase in the cell cycle time and a large proportion of cells which are " out of cycle " (Hahn and Little, *Curr. Topics Radiat. Res.*, 1972, **8**, 39). There is therefore a similarity between stationary phase cultures and solid tumours which also contain "non-cycling " cells.

Dose-response curves are presented for the effect of a number of chemotherapeutic agents on EMT6 mouse tumour cells in exponential and stationary phases of growth. The similarities and differences between our results and those of other workers are discussed.